# The Role of hsa-miR-125b-5p Interaction with S1P/Ceramide Axis in the Potential Development of Inflammation-Associated Colon Cancer in Primary Sclerosing Cholangitis

**DOI:** 10.3390/ijms24119175

**Published:** 2023-05-24

**Authors:** Joanna Abramczyk, Malgorzata Milkiewicz, Bartosz Hula, Piotr Milkiewicz, Agnieszka Kempinska-Podhorodecka

**Affiliations:** 1 Department of Medical Biology, Pomeranian Medical University, 70-111 Szczecin, Poland; joanna.abramczyk@pum.edu.pl (J.A.); malgorzata.milkiewicz@pum.edu.pl (M.M.); bartoszhulapum@gmail.com (B.H.); 2 Liver and Internal Medicine Unit, Medical University of Warsaw, 02-097 Warsaw, Poland; p.milkiewicz@wp.pl; 3 Translational Medicine Group, Pomeranian Medical University, 70-111 Szczecin, Poland

**Keywords:** microRNA, microsatellite instability, cholestatic liver disease, inflammatory bowel disease, colorectal cancer

## Abstract

Primary sclerosing cholangitis (PSC) is characterised by the co-occurrence of inflammatory bowel diseases, particularly ulcerative colitis (UC). We investigated how the interaction of miR-125b with the sphingosine-1-phosphate (S1P)/ceramide axis may predispose patients with PSC, PSC/UC, and UC to carcinogenesis in the ascending and sigmoid colons. The overexpression of miR-125b was accompanied by the upregulation of S1P, ceramide synthases, ceramide kinases, and the downregulation of AT-rich interaction domain 2 in the ascending colon of PSC/UC, which contributed to the progression of high microsatellite instability (MSI-H) colorectal carcinoma. We also showed that the overexpression of sphingosine kinase 2 (SPHK2) and the genes involved in the glycolytic pathway in the sigmoid colon of UC led to the upregulation of Interleukin 17 (IL-17). In vitro stimulation of human intestinal epithelial cells (Caco-2, HT-29, and NCM460D) with lipopolysaccharide suppressed miR-125b and increased proinflammatory cytokines, whereas the induction of miR-125b activity by either a miR-125b mimetic or lithocholic acid resulted in the inhibition of miR-125b targets. In summary, miR-125b overexpression was associated with an imbalance in the S1P/ceramide axis that can lead to MSI-H cancer progression in PSC/UC. Furthermore, SPHK2 overexpression and a change in the cellular metabolic flux are important players in inflammation-associated colon cancer in UC.

## 1. Introduction

Cholestatic liver diseases, such as primary sclerosing cholangitis (PSC), have complex aetiologies and are characterised by the progressive destruction of liver structures, via cholestasis and autoimmunity [1]. One disorder that commonly accompanies PSC is ulcerative colitis (UC). PSC may be an important risk factor for colorectal cancer (CRC) in inflammatory bowel disease (IBD). Importantly, the majority of CRCs in PSC patients are located on the right side of the colon (ascending colon), unlike in patients with UC alone, where the tumours more frequently occur on the left side (sigmoid colon). The risk of CRC developing in PSC/UC patients is believed to be 4–10 times greater than in patients with only UC, and it develops at a much younger age than in patients with UC alone [2].

Colorectal carcinoma is not a uniform disease and can be distinguished by a range of genomic and epigenomic modifications [3]. A deficiency in the mismatch repair pathway has been known to induce microsatellite instability (MSI), and an accumulation of DNA replication errors—particularly in areas with short repetitive nucleotide sequences [4]. Exome sequencing has uncovered frequent inactivating mutations in the AT-rich interaction domain 2 (ARID2) in microsatellite unstable colorectal cancer. The ARID proteins participate in various, central biological processes, such as embryonic patterning, cell lineage gene regulation, cell cycle control, transcriptional regulation, and chromatin remodelling [5]. The mechanism of CRC tumour I genesis has also been linked to miRNAs [6]. These are a class of small (~20 nucleotides), endogenous, non-coding RNAs that modulate gene expression by binding to the 3′-UTR of the target mRNA, leading to either its degradation or repression of protein translation. Indeed, miRNAs may possess either tumour-suppressive or oncogenic activity, depending on their target genes [7]. The important role of miRNAs in the immune response is highlighted by studies in which the deregulation of miRNAs has been shown to accompany diseases involving excessive or uncontrolled inflammation [8]. However, research into the role of miRNAs in the pathogenesis of susceptibility to colon carcinogenesis in patients suffering from cholestatic liver diseases, such as PSC, is insufficient. In the context of bowel disease, it has been shown that expression of miR-125b is significantly higher in inflamed mucosa compared to non-inflamed regions of UC patients and controls [9]. The controversial properties of miR-125b as an oncogene or tumour suppressor in different solid tumours suggest that miR-125 plays diverse roles in cancer pathogenesis and progression [10,11,12]. However, to date, the functions of miR-125b in the pathogenesis of CRC in PSC remain unclear.

A growing body of literature suggests that the sphingosine 1-phosphate (S1P)/ceramide axis plays a role in cancer and in inflammation-associated tumours. The accumulation of sphingolipids accounts for nearly 90% of the changes observed in CRC cell lines [13]. They are fundamental components of cell membranes and their metabolites, namely ceramide, sphingosine, S1P, and ceramide-1-phosphate (C1P), play essential, yet opposite roles in the growth and death of mammalian cells. Ceramide has been implicated as an antiproliferative and proapoptotic messenger, whereas S1P and C1P promote cellular proliferation and survival [14,15].

The development and progression of CRC are accompanied by alterations in the sphingolipid composition in colonic tumours [16]. S1P is a bioactive sphingolipid and a cellular signalling molecule that inhibits apoptosis, promotes oncogenesis, and augments inflammation, via nuclear factor kappa-light-chain-enhancer of activated B cells (NF-κB)related pathways [17]. Chronic, but not acute, inflammatory signals increase the expression of both the S1P receptor (S1PR1) and the enzymes that regulate the S1P levels [18]. The enzymes that control tissue S1P levels are uniformly dysregulated in mice and humans with IBD, favouring synthesis over degradation. The strategy of blocking T-cell egress through the administration of FTY720 (a sphingosine analogue) has shown benefits in the treatment of patients with autoimmune diseases and IBD [19].

S1P can be irreversibly degraded by sphingosine lyase (SPL), while sphingosine kinases (SPHKs) catalyse the synthesis of S1P from sphingosine. The SPHK/S1P signalling pathway is associated with cancer development and metastasis [20]. In the salvage pathway, hydrolytic enzymes, including sphingosine-1-phosphate phosphatase (SGPP) produce sphingosine. The resultant sphingosine can be reused for the synthesis of ceramide. Ceramide synthases (Cers) are enzymes involved in the de novo synthesis of ceramides from sphingoid bases [21], and the phosphorylated metabolite of ceramide (C1P) is a regulator of cancer cell proliferation and migration [22,23,24].

Our unpublished microarray data indicate that miR-125 is upregulated in the livers of PSC patients. In previous studies, S1PR1 [25], SPHK1 [26], and SPL [27] were identified as direct targets of miR*-*125b. Given that: (i) miR-125b is directly involved in the progression of colorectal cancer; (ii) CRC is accompanied by alterations in the sphingolipid composition in colon tumours; (iii) SGPP1 promotes disruption of mucosal integrity; (iv) SPHK2 promotes the progression of inflammation by inducing IL-17, we investigated the involvement of miR-125 and the S1P pathway in pathological changes that may predispose patients with primary sclerosing cholangitis to carcinogenesis of colonic tissue.

## 2. Results

### 2.1. miR-125b-Related Signalling Pathways

**Figure 1:**
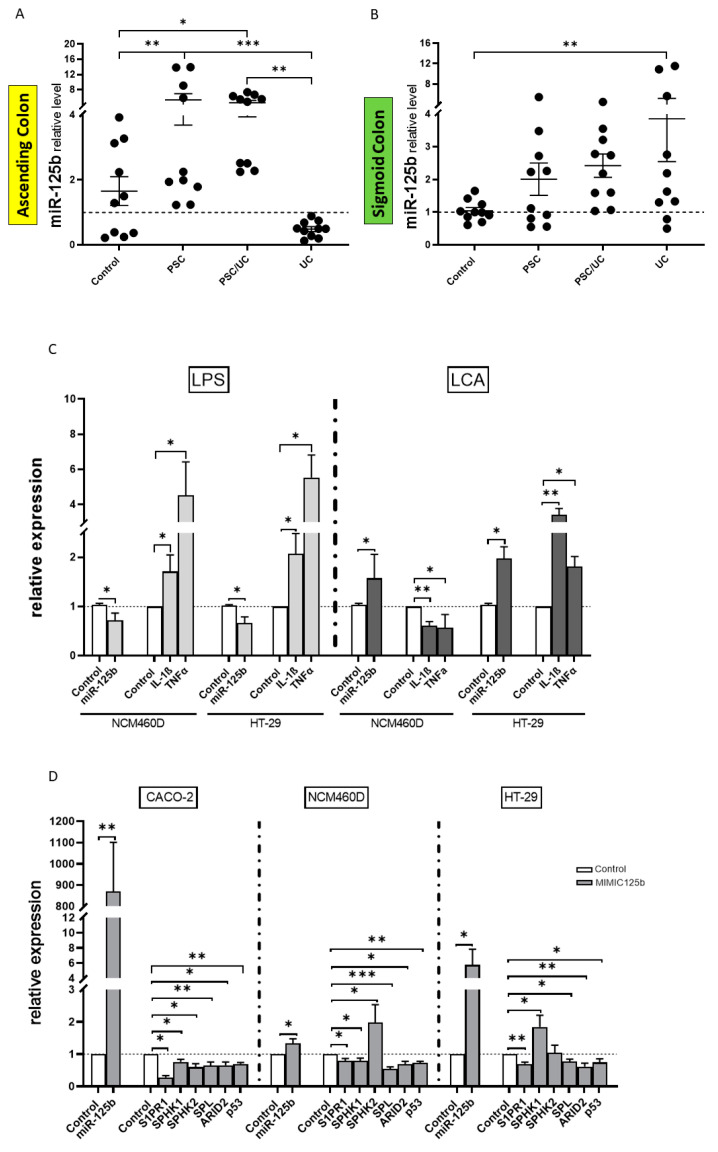
The expression of miR-125b in colonic tissue and patterns of miR-125b-related signalling pathways in three colonic cell lines (Caco-2, NCM460D, and HT-29). A scatter dot plot showing the relative expression levels of miR-125b in the ascending (**A**) and sigmoid colons (**B**) of controls, primary sclerosing cholangitis (PSC) patients, PSC, and concomitant ulcerative colitis patients (PSC/UC) and ulcerative colitis (UC). The effect of lipopolysaccharide (LPS) and lithocholic acid (LCA) on interleukin-1 beta (1L-1β) and tumour necrosis factor-α (TNFα) expression, via miR-125b, in colonic cell lines following 24 h treatment (**C**). Modulation of the sphingosine-1-phosphate receptor (S1PR1), sphingosine kinase 1 (SPHK1), SPHK2, sphingosine lyase (SPL), AT-rich interaction domain 2 (ARID2) and p53 gene expression levels after miR-125b mimetic transfection in colonic cell lines (**D**). At least three independent experiments were conducted. The dotted lines represent control values. Levels of gene expression were normalised to the endogenous reference miR-16 for miRNA or 18S RNA for other genes. Bars indicate the mean ± SEM. * *p*-value < 0.05, ** *p*-value < 0.01, *** *p*-value < 0.001.

In the ascending colon, the expression of miRNA-125 was substantially increased in PSC without UC (3-fold increase vs. controls, *p* = 0.007; and an 11-fold increase vs. UC, *p* = 0.0006) and PSC with UC (3-fold increase vs. controls, *p* = 0.03; and 9-fold increase vs. UC, *p* = 0.003) (Figure 1A). Additionally, the miR-125b level was enhanced in the sigmoid colon of the UC patients (3-fold increase vs. controls, *p* = 0.009) (Figure 1B). This suggests differences in inflammation in the sigmoid colon of PSC/UC patients in comparison to patients with UC alone.

The biological relevance of miR-125b upregulation in the context of inflammatory bowel disease was evaluated in lipopolysaccharide (LPS) stimulated human intestinal epithelial cells. LPS-stimulation caused a marked inhibition of miR-125b (vs. controls, *p* = 0.05 and *p* = 0.02, respectively) and was associated with the activation of interleukin-1 beta (IL-1β) (vs. controls, *p* = 0.05 and *p* = 0.04, respectively) and tumour necrosis factor-α (TNFα) (vs. controls, *p* = 0.02 and *p* = 0.01, respectively) genes in normal colonic mucosa (NCM460D) and in the cancerous cell line HT-29 (Figure 1C).

To gain insight into the effects of bile acid on miR-125b in the intestine, the expression of miRNA was measured following lithocholic acid (LCA) exposure in both enterocyte lines (Figure 1C). Secondary bile acid (LCA: 100 µM) markedly increased miR-125b levels in NCM460D (vs. controls, *p* = 0.04) and HT-29 (vs. controls, *p* = 0.02) cells after 24 h of exposure. LCA-induced upregulation of miR-125b suppressed IL-1β (vs. controls, *p* = 0.003) and TNFα (vs. controls, *p* = 0.03) mRNA in NCM460D cells, whereas in HT-29, those cytokines expressions were substantially enhanced (vs. controls, *p* = 0.003 and *p* = 0.02 vs. controls, respectively), despite the overexpression of miR-125b (Figure 1C). LCA-induced upregulation of miR-125b did not induce cytokine expression as the NCM460D cells we observed suppressed IL-1β and TNFα mRNA, whereas in HT-29 those cytokines were induced (Figure 1C). Furthermore, we detected miR-125b targets involved in tumorigenesis. We showed that transfection of miR-125b mimetics into Caco-2, NCM460D, and HT-29 cell lines led to the direct inhibition of S1PR1, SPL, ARID2, and p53 mRNA (Figure 1D). A higher expression of SPHK1 was observed in the cancerous cell line HT-29, whereas SPHK2 was induced in NCM460D (Figure 1D).

### 2.2. SPHKs/S1P Signalling Axis in Inflammation

**Figure 2:**
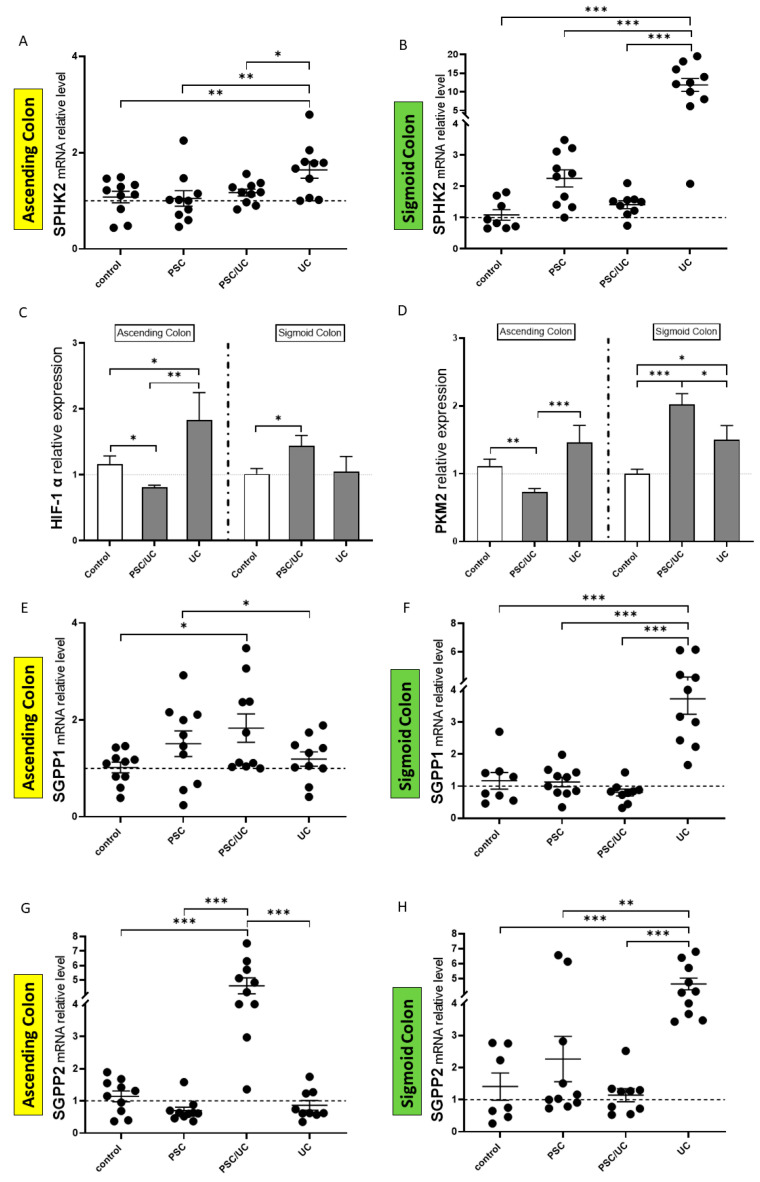
The expression of enzymes involved in S1P metabolism and levels of crucial regulators of immunometabolism. Scatter dot plots show the relative expression of SPHK2, sphingosine-1-phosphate phosphatase 1 (SGPP1), and SGPP2 mRNA in the ascending (**A**,**E**,**G**), and sigmoid colon (**B**,**F**,**H**) controls, PSC, PSC with UC, and UC patients. The expression of hypoxia-inducible factor 1α (HIF-1α) and pyruvate kinase M2 (PKM2) mRNAs in the ascending (**C**) and sigmoid colon (**D**) of PSC/UC and UC are presented as bar charts. Levels of gene expression are presented as fold-changes relative to healthy controls and were normalised to the endogenous reference 18S RNA. The dotted lines represent control values. Results are representative of 10 independent experiments per group. Dots illustrate each patient and data are presented as means plus interquartile ranges (IQRs). * *p*-value < 0.05, ** *p*-value < 0.01, *** *p*-value < 0.001.

SPHK2 expression increased in both the ascending and sigmoid colons of UC patients. In the ascending colon, it was significantly enhanced in UC compared to controls, PSC, and PSC/UC (Figure 2A). Similarly, the level of SPHK2 mRNA was induced in the sigmoid colon of PSC and PSC/UC (Figure 2B). As SPHK2 expression in human T cells has been reported to be modulated by IL-17 [28], we calculated correlations between SPHK2 and IL-17a mRNA in the sigmoid colon of UC (our previously published date) [29]. In this study, we observed a strong positive correlation between SPHK2 and IL-17a mRNA in the colonic tissue of UC patients (r = 0.7, *p* = 0.0009).

This inflammation could induce metabolic reprogramming and promote the progression from chronic colitis to colorectal cancer [30]. Therefore, we investigated the regulators of immunometabolism, including hypoxia-inducible factor 1 α (HIF-1α) and the glycolytic enzyme pyruvate kinase M2 (PKM2) in patients with PSC/UC and UC (Figure 2C,D). We observed a positive correlation between HIF-1α and PKM2 in the sigmoid colon of patients with PSC/UC (r = 0.4, *p* = 0.05) and in both parts of the colons of UC patients (r = 0.7, *p* = 0.001 and r = 0.5, *p* = 0.02 in the ascending and sigmoid colons, respectively). In contrast, the level of HIF-1α and PKM2 were downregulated in the ascending colon of patients with PSC (r = 0.7, *p* = 0.001).

Due to the activities of SPHKs, both sphingosine and S1Ps were in dynamic equilibrium. In the ascending colon of PSC/UC, both SGPP1 and SGPP2 mRNA expressions were upregulated (1.8-fold increase vs. controls, *p* = 0.01; and a 4.4-fold increase vs. controls, *p* = 0.0001, respectively) (Figure 2E,G). In addition, the relative levels of SGPP1 mRNA in PSC patients were 30% higher than in UC patients (Figure 2E). Furthermore, we observed the upregulation of SGPP2 mRNA in the ascending colons of PSC/UC in comparison to PSC and UC alone (6.6-fold increase vs. PSC, *p* = 0.0001; 5.3-fold increase vs. UC, *p* = 0.0001, respectively), (Figure 2G). In contrast, the levels of SGPP1 and SGPP2 mRNA were not changed in the sigmoid colon of PSC and PSC/UC patients in comparison to the controls (Figure 2F,H). In patients with UC alone, the expression of SGPP1 was substantially enhanced (3.1*-*fold increase vs. controls, *p* = 0.0001*,* a 3.4*-*fold increase vs. PSC, *p* = 0.0001, and a 4.6*-*fold increase vs. PSC/UC, *p* = 0.0001) (Figure 2F). Similarly, induction of SGPP2 expression was observed in UC sigmoid colons (3.3*-*fold increase vs. controls, *p* = 0.0001, a 2*-*fold increase vs. PSC, *p* = 0.001, and a 4.2*-*fold increase vs. PSC/UC, *p* = 0.0001), (Figure 2H).

### 2.3. The Expression of Enzymes Involved in the Ceramide Pathway

**Figure 3:**
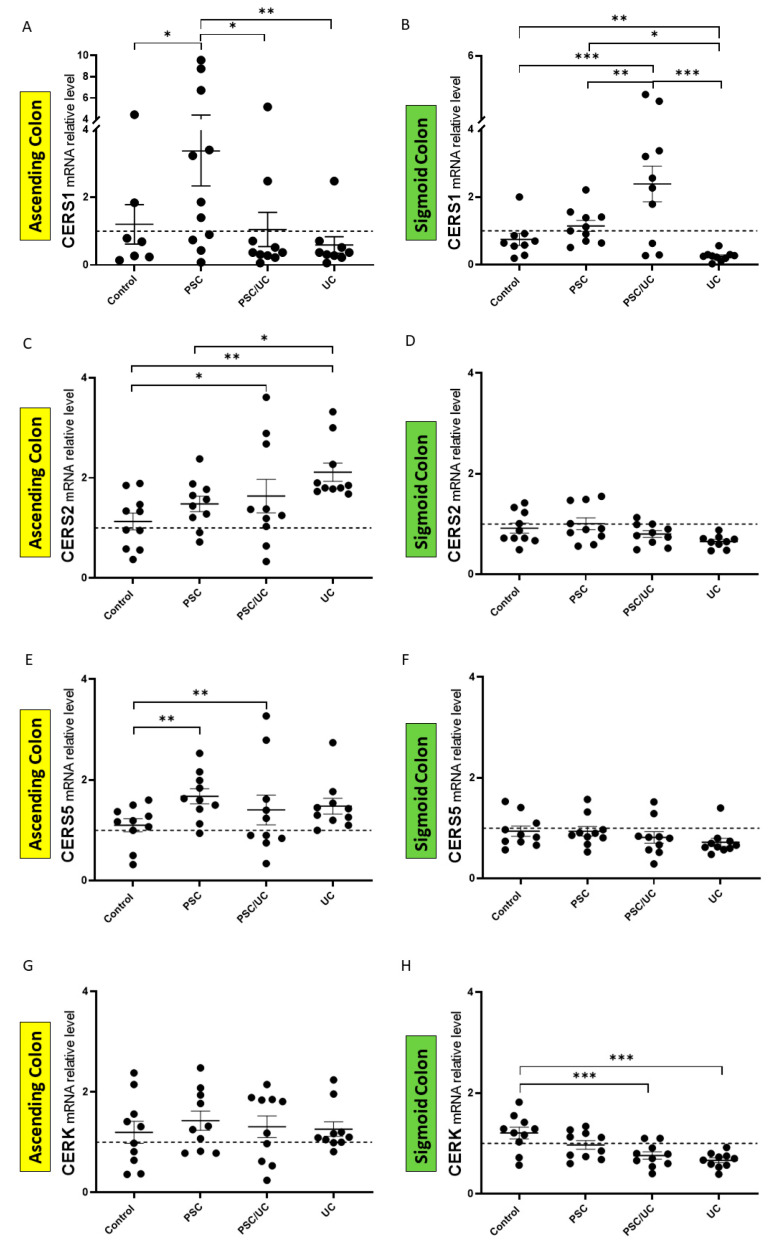
The expression of enzymes implicated in ceramide-1-phosphate (C1P) metabolism. Scatter dot plots show the relative expression levels of ceramide synthase 1 (CERS1), CERS2, CERS5, and ceramide kinase (CERK) mRNA in the ascending colon (**A**,**C**,**E**,**G**) and sigmoid colon (**B**,**D**,**F**,**H**) in the controls, PSC, PSC with UC, and UC patients. Levels of gene expression are presented as fold-changes relative to healthy controls and were normalised to the endogenous reference 18S RNA. The dotted lines represent control values. Results are representative of 10 independent experiments per group. Dots illustrate each patient and data are presented as means plus interquartile ranges (IQRs). * *p*-value < 0.05, ** *p*-value < 0.01, *** *p*-value < 0.001.

In the ascending colon of PSC without UC, the expression of Cers1 mRNA was upregulated (vs. controls, *p* = 0.01, vs. PSC/UC, *p* = 0.01, and vs. UC, *p* = 0.002) (Figure 3A). In the sigmoid colon of PSC/UC, the overexpression of Cers1 mRNA was observed (vs. controls, *p* = 0.0005, vs. PSC, *p* = 0.005, and vs. UC, *p* = 0.0001) (Figure 3B). Furthermore, we observed significant Cers1 mRNA downregulation in the sigmoid colon of UC (vs. control, p = 0.004 and vs. PSC, p = 0.04). The overexpression of Cers2 was observed in the ascending colon of PSC/UC (vs. controls, *p* = 0.02) and UC (vs. controls, *p* = 0.002 and vs. PSC, *p* = 0.03) (Figure 3C). The upregulation of Cers5 mRNA was observed in the ascending colon of PSC patients (1.6-fold increase vs. controls, *p* = 0.007) and PSC/UC (1.6-fold increase vs. controls, *p* = 0.005) (Figure 3E). In the sigmoid colon of all patients, the levels of Cers2 and Cers5 were unchanged (Figure 3D,F) and in the ascending colon of all patients, the level of ceramide kinase (CERK) was unchanged (Figure 3G). The downregulation of CERK expression was observed in the sigmoid colon of PSC/UC (*p* = 0.0006) and UC (*p* = 0.0001) patients (Figure 3H). These results suggest that CERK catalyses the synthesis of ceramide-1-phosphates from ceramides and is dysregulated in the sigmoid colon of patients with inflammatory bowel disease.

### 2.4. LCA-Induced Regulation of the Ceramide Pathway

**Figure 4:**
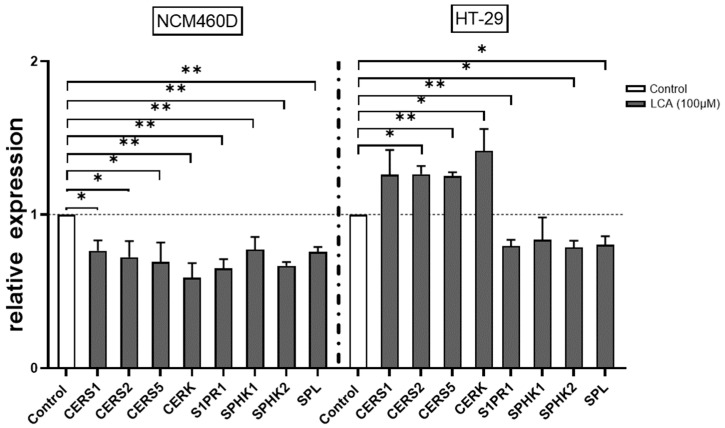
The effect of LCA on the regulation of the ceramide pathway in normal colonic mucosa (NCM460D) and a cancerous cell line (HT-29). CERS1, CERS2, CERS5, CERK, S1PR1, SPHK1, SPHK2, and SPL gene mRNA levels in both colonic cell lines following LCA exposure. At least three independent experiments were conducted. The dotted lines represent control values. Levels of gene expression were normalised to the endogenous reference 18S RNA. Bars indicate the mean ± SEM. * *p*-value < 0.05, ** *p*-value < 0.01.

Given that LCA, an established tumour promoter, has been implicated in CRC metastasis [31] and is primarily presented within the colon [32], we examined the expression of Cers1, Cers2, Cers5, and CERK in LCA-stimulated human intestinal epithelial cells. We found that in response to LCA, the levels of Cers1, Cers2, Cers5, and CERK varied between cancerous cell lines and normal colonic mucosa cells (Figure 4A). LCA treatment suppressed the expression of ceramide synthases and ceramide kinase in HT-29 cells, whereas, in NCM460D cells, it induced Cers1, Cers2, Cers5, and CERK expression. Additionally, LCA effectively reduced the expression of S1PR1, SPHK1, SPHK2, and SPL in both intestinal epithelial cells (Figure 4A).

### 2.5. ARID2 in Microsatellite-Unstable (MSI) Colorectal Cancer

**Figure 5:**
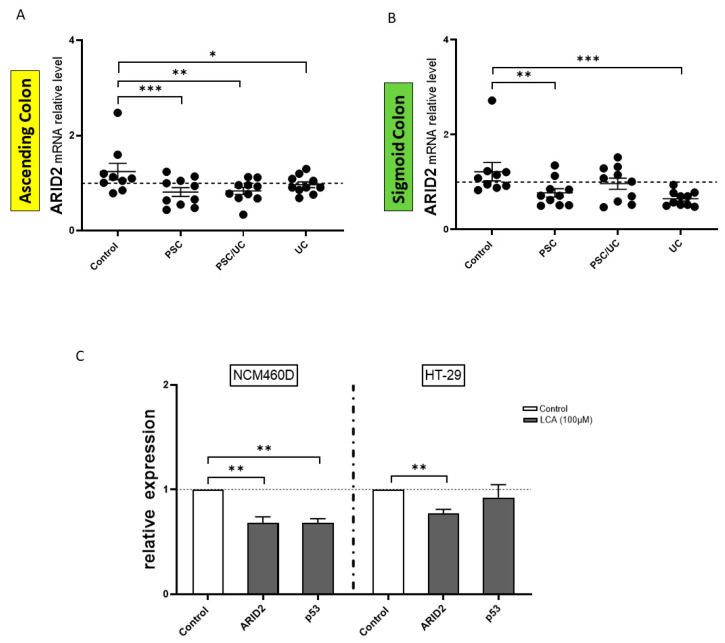
The expression of ARID2 mRNA in colonic tissue and colonic cell lines (NCM460D and HT-29). A scatter dot plot showing the relative expression levels of ARID2 in the ascending (**A**) and sigmoid (**B**) colons of the controls, PSC, PSC/UC, and UC patients. Modulation of ARID2 and p53 gene expression levels in both cell lines following 24 h LCA treatment (**C**). Levels of gene expression were normalised to the endogenous reference 18S RNA. The dotted lines represent control values. Bars indicate the mean ± SEM. * *p*-value < 0.05, ** *p*-value < 0.01, *** *p*-value < 0.001.

MiR-125b is associated with the modulation of ARID2. In the ascending colon of all patients, a substantial downregulation of ARID2 expression was observed (PSC: 40% reduced vs. controls, *p* = 0.003; PSC/UC: 40% reduced vs. controls, *p* = 0.004; and UC: 38% reduced vs. controls, *p* = 0.04; Figure 5A). In the sigmoid colon of the PSC/UC patients, the ARID2 levels were similar to the control values, whereas in PSC and UC patients the expression of ARID2 mRNA was downregulated (40% reduced vs. controls, *p* = 0.006; and 54% reduced vs. controls, *p* = 0.0008, respectively)(Figure 5B). Additionally, LCA was associated with the downregulation of ARID2 mRNA in NCM460D (vs. controls, *p* = 0.002) and HT-29 (vs. controls, *p* = 0.008) cells (Figure 5C). In NCM460D cells, the expression of p53 mRNA was downregulated (vs. controls, *p* = 0.003), whereas in *HT-29 cells* the expression of p53 mRNA was similar to the controls. These results indicate that the ARID2 motif is likely to be important for the pathogenesis of colorectal cancer in the ascending colon of PSC/UC.

## 3. Discussion

This research highlights points of connection between miR-125b and sphingolipid metabolism in colonic mucosa and shows that an imbalance in the S1P/ceramide axis, via miR-125b, can lead to high microsatellite instability (MSI-H) cancer progression in the ascending colon of patients with PSC/UC. Furthermore, the overexpression of SPHK2 and a shift of metabolites involved in energy metabolism (the Warburg effect) are important players in inflammation-associated colon cancer progression in the sigmoid colon of UC.

The overexpression of miR-125 was observed in the ascending colon of PSC/UC and in the sigmoid colon of UC. Our results regarding the increase in miR-125b expression in the sigmoid colon of UC patients are in line with other studies [33]. MiR-125b upregulation is associated with advanced tumour stages and invasion of local tissues [10]; however, the underlying mechanism is unknown. Given that S1P and C1P are involved in inflammation and cancer, we focussed on understanding the role of the S1P/ceramide axis in PSC linked to CRC.

Firstly, we focussed on the role of the S1P axis in inflammation and cancer. We investigated the expressions of enzymes involved in S1P turnover and the signalling pathways of S1P metabolites in human colonic tissue of PSC/UC and UC. Generally, the cellular S1P concentration is regulated by the balance between its dephosphorylation, which is mediated by SGPPs, and its synthesis by sphingosine kinases [16]. In this study, we detected the overexpression of S1P phosphatases, such as SGPP1 and SGPP2 mRNAs, in the ascending colon of PSC/UC and in the sigmoid colon of the UC. These observations are in agreement with previous reports showing a significant increase in SGPP2 expression in the sigmoid colons of UC patients and in the sigmoid colons of twins with UC compared with their healthy siblings [34]. Little is known about the physiologic functions of S1P phosphatases, although it was reported that SGPP1 deletion enhanced the expression of multifunctional, proinflammatory cytokines, and immune cell infiltration into the colon, whereas Sgpp2 deficiency suppressed intestinal epithelial cell SGPP apoptosis and improved the mucosal barrier integrity in mice [34].

We observed the induction of SPHK2, although only in the sigmoid colon of UC patients. SPHK2 promotes the progression of inflammation by enhancing the biosynthesis of IL-17 [28], a marker for Th17 cells. In this study, we confirmed the correlation between SPHK2 and IL-17 in the sigmoid colon of UC patients. Thus, our results suggest that, in contrast to PSC/UC patients, the upregulation of the SPHK2/S1P axis may be responsible for chronic intestinal inflammation and could promote the transformation to dysplastic changes in UC patients. A switch of metabolic flux in immune cells from oxidative phosphorylation to aerobic glycolysis (the Warburg effect) is a likely mechanism behind sustained inflammation [35]. Interestingly, the upregulation of HIF-1α and PKM2 in precancerous colorectal lesions compared to normal controls occurs even in premalignant tissue [36]. We established that HIF-1α and PKM2 are upregulated in the sigmoid colon of patients with UC. PKM2 levels in serum and faeces are elevated in IBD, including in UC patients [37,38]. It is worth mentioning that these factors were suppressed in the ascending colon of PSC/UC, which may suggest a different pathogenesis of CRC in PSC/UC patients than in those with UC alone.

Secondly, knowing that lipid mediators, such as C1P, may contribute to both inflammation and cancer [39,40,41], we investigated whether the C1P axis is involved in inflammation-associated cancer. We found that, in contrast to UC alone, ceramide synthases such as Cers2 and Cers5 were upregulated in the ascending colon of PSC/UC patients. Accumulating evidence suggests that the overexpression of Cers2 leads to increased cell proliferation in colon cancer cell lines [42], while high Cers5 expression has been found in colorectal cancer tissue and is associated with poorer patient outcomes [43]. Furthermore, the strong suppression of CERK, the enzyme that metabolises ceramide to antiapoptotic C1P, was noticed in the sigmoid of UC but not in the ascending colon of PSC/UC patients [44]. Our in vitro functional studies showed that exposure to enhanced concentrations of bile acids can contribute to the development of tumorigenic transformation. Bile acid, namely LCA, activated cancer-promoting genes including Cers1, Cers2, Cers5, and CERK in the cancerous HT-29 cell line in a miR125b-dependent way.

MiR-125b is known to enhance cancer progression by targeting p53 [45]. In our previous study, we observed the downregulation of p53 mRNA in the ascending colon of PSC/UC patients [46]. In this study, we demonstrated that increased miR-125b expression was associated with the downregulation of ARID2 in the same tissue. ARID, a novel tumour suppressor gene that positively regulates double-strand break repair may play an essential role in human cancers and, when mutated, contribute to MSI CRC development [47]. Moreover, ARID2 depletion promotes CRC cell proliferation and inhibits apoptosis by regulating the activity of the Akt signalling pathway [48]. A low level of ARID was closely associated with larger tumour size, right-sided tumours, and a high histological grade of CRC [49]. The significant association between ARID deletions and mismatch repair (MMR) defects in CRC has been demonstrated, and the loss of ARID expression was found in 15–25% of MMR-deficient (vs. 4–6% of MMR-intact) CRC cases [50]. Of note, we previously reported that the suppression of MMR proteins may be responsible for the initiation of colorectal neoplastic transformations in patients with PSC [29]. In agreement with earlier reports [49], and in line with our recent study [29], we suggest that ARID2 and p53 alterations modulated by miR-125b could be responsible for molecular changes that prompt MSI-H CRC in the ascending colon of PSC/UC. Our in vitro analysis confirmed that bile acids, for which concentrations are enhanced in the colons of PSC patients, raise the level of miR-125b in human intestinal epithelial cells. Enhanced expression of LCA effectively reduces the expression of ARID2 in both cell lines and p53 in normal epithelial cells. Additionally, it induces the expression of TNF-α and IL-1β in cancerous cell lines, yet not in healthy enterocyte cell lines. It has also been shown that perturbations in the plasma membrane by BA activate the NF-κB axis [51]. Activated NF-κB translocates into the nucleus, where it transcribes the gene encoding IL-1β [52], which activates the PI3K–MDM2 axis, resulting in a blockage of p53 activity. Inhibition of p53 leads to the suppression of apoptosis and to the enhanced survival of DNA-damaged cells, which contributes to potential CRC development [52,53]. Our in vitro analysis confirmed that the experimentally induced overexpression of miR-125b efficiently suppressed ARID2, p53, and SPL genes in both healthy and tumorous intestinal enterocytes. However, we did not observe miR125b-dependent modulation of SPHK2 in those cell lines. Thus, our findings imply that the pathogenesis of CRC in patients with PSC/UC may be modulated by miR-125b and its target genes.

## 4. Materials and Methods

### 4.1. Subjects

We included four groups of patients from whom colonic tissue samples were collected. The study included patients with PSC who underwent routine surveillance colonoscopies. Based on a histopathological analysis of colon tissue performed by a pathologist, the patients were divided into two groups: (i) PSC patients (*n* = 10) who had never been diagnosed with concomitant inflammatory bowel disease and (ii) PSC/UC patients (*n* = 10) exhibiting the macroscopic features of UC on colonoscopy, which were confirmed with a histology examination. Additionally, patients with (iii) active UC (*n* = 10) and (iv) healthy controls who underwent colonoscopies for various indications and showed neither macroscopic nor microscopic abnormalities in their colons (*n* = 10) were included in the study.

Colon biopsy specimens were obtained from the ascending and sigmoid colons of each patient. Inclusion criteria for the study were: patients fulfilling EASL criteria for primary sclerosing cholangitis; age: between 18 and 75 years; standard treatment (5-aminosalicylic acid and azathioprine). Exclusion criteria: an inability to give informed consent; patients with other forms of chronic liver and colon diseases; the presence of decompensated liver cirrhosis (ChildPugh class B–C); steroid therapy; pregnancy or breastfeeding; any other condition that, in the opinion of the investigators, would impede the patient’s participation or compliance in the study.

Demographic and clinical data of the analysed patients are summarised in Table 1. Each patient gave informed consent prior to participating in the study. The research protocol was approved by the Ethics Committee of Pomeranian Medical University (KB001/43/06 and KB0012/44/2021) and conformed to the ethical guidelines of the 1975 Declaration of Helsinki.

### 4.2. Cell Culture Transfection and Treatments

NCM460D (INCELL Innovative Life Science Solutions, San Antonio, TX, USA; Cell License Material Transfer Agreement #204), representing a mixed monolayer suspension culture that was spontaneously immortalized and non-tumorigenic, was used as a model of colonic epithelium. Cells were grown in INCELL’s enriched M3:10 Base F Medium (INCELL Innovative Life Science Solutions San Antonio, TX, USA), 10% *v*/*v* foetal bovine serum (ATCC 30-2025), and 1% antibiotic solution (penicillin/streptomycin; Biowest, Nuaille, France), according to the manufacturer’s recommendations. Caco-2 (HTB-37™) are immortalized heterogeneous human epithelial colorectal adenocarcinoma cells, and HT-29 (HTB-38™) is a colorectal adenocarcinoma cell line with epithelial morphology. Both were purchased from the American Type Culture Collection and were grown according to the original protocol. All of the cell lines were cultured in 25 cm^2^ or 75 cm^2^ culture flasks and routinely maintained in a humidified atmosphere of 5% CO_2_ at 37 °C.

Transient transfections with miR-125b mimic (Ambion mirVana^®^miRNA mimic, hsa-miR-125b; ID: MC10148; Thermo Fisher Scientific, Waltham, MA, USA) were performed using Lipofectamine RNAiMAX reagent (Invitrogen, Carlsbad, CA, USA), in accordance with the manufacturer’s protocol. Forty-eight hours after transfection of CaCO_2_, or seventy-two hours for NCM460D and HT-29, the cells were lysed, and RNA was isolated for further analysis.

To investigate the effect of LCA on miR-125b expression, NCM460D and HT-29 cells were exposed to 100 µM of LCA for 24 h. LCA, provided by Sigma (St. Louis, MO, USA), was dissolved in sterile dimethyl sulfoxide.

To initiate the inflammatory process, NCM460D and HT-29 cells were incubated in an appropriate, complete cell culture medium with the addition of LPSs from *Escherichia coli* 0111:B4 (0.5 µg/mL) (4391, Sigma, St. Louis, MO, USA). After 24 h, the cells were lysed, and RNA was isolated for further analysis.

### 4.3. RNA and miRNA Expression Analysis

Total RNA was isolated from human colon tissues and epithelial cells using RNeasy Mini kit (Qiagen, Hilden, Germany), and cDNA synthesis was performed using the TaqMan Advanced miRNA cDNA Synthesis Kit (Applied Biosystems, Thermo Fisher Scientific, Waltham, MA, USA) or SuperScript IV RT (Invitrogen, Carlbad, CA, USA), based on the manufacturer’s protocol. Gene expressions were analysed using human TaqMan Gene Expression Assays for 18S ribosomal RNA (Hs99999901_s1), S1PR1 (Hs01922614_m1), IL-17A (Hs00174383_m1), VDR (Hs00172113_m1), IL-1β (Hs01555410_m1), TNFα (Hs00174128_m1), SPKH1 (Hs00184211_m1), SPHK2 (Hs01016543_g1), SPL (Hs00393705_m1), SGPP1 (Hs00229266_m1), SGPP2 (Hs00544786_m1), Cers1 (Hs04195319_s1), Cers2 (Hs00371958_g1), Cers5 (Hs00332291_m1), CERK (Hs00968483_m1), ARID2 (Hs00326029_m1), and p53 (Hs01034249_m1) cDNA synthesis was conducted using the TaqMan Advanced miRNA cDNA synthesis kit (Applied Biosystems, Waltham, MA, USA), according to the manufacturer’s protocol. The expression of miR-125b (477885_mir) and reference microRNA miR-16 (477860_mir) were measured using TaqMan Advanced miRNA assays and Taq Man Fast Advanced Master Mix (Applied Biosystems, Waltham, MA, USA). Fluorescence data were analysed using 7500 software v2.0.2. (Applied Biosystems, Waltham, MA, USA) and the relative amounts of transcripts were calculated using the 2^−∆∆Ct^ formula.

### 4.4. Statistics

Statistical analyses were conducted using StatView (SAS Institute, Cary, NC, USA) and GraphPad Prism 9.5.0 applications (GraphPad Software, San Diego, CA, USA). Continuous variables are shown as mean ± SEM. Statistical differences in baseline characteristics between groups were analysed using the x^2^ test for categorical data and the Mann–Whitney U test or Student’s *t*-test for quantitative data. Correlation analyses were performed using Spearman’s rank method. Results were considered statistically significant when *p*-values were <0.05.

## 5. Conclusions

**Figure 6:**
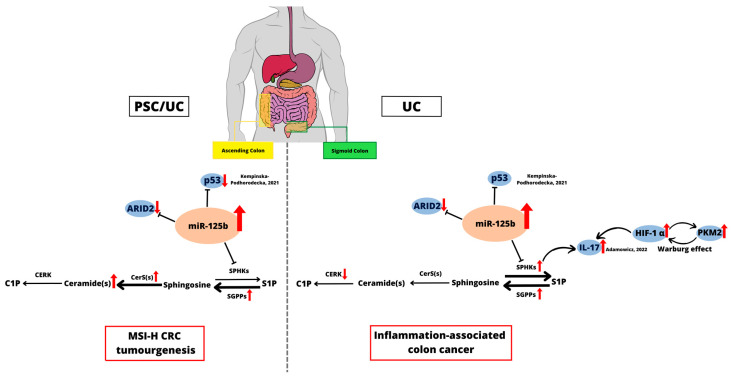
Schematic drawing showing the connection points between miR-125b and sphingolipid metabolism in the colonic mucosa. The interaction of miR-125b with p53 [46], ARID2 and imbalance in S1P/ceramide axis may lead to MSI-H cancer progression in the ascending colon in PSC/UC patients. In addition, overexpression of both IL-17a [29] and SPHK2 along with alterations in the cellular metabolic flux are important factors that may predispose to inflammation-associated colon cancer in UC.

The overexpression of miR-125b was found to be directly associated with an imbalance in the S1P/ceramide axis and the potential development of MSI-H CRC in the ascending colon of PSC patients with concomitant UC (Figure 6). In contrast, tumour-promoting inflammation in the sigmoid colon of UC depended on the overexpression of SPHK2. We suggest that selective modulation of miR-125 may be a potential target for future pharmacological interventions, including molecular targeted therapy for MSI-H CRC in PSC/UC.

## Figures and Tables

**Table 1:** Demographic and laboratory features of subjects who underwent analysis.

	Control (*n* = 10)	PSC (*n* = 10)	PSC/UC (*n* = 10)	UC (*n* = 10)
**Gender (*Male/Female*)**	6/4	8/2	8/2	2/8
**Age (*years*)**	58 ± 4	44 ± 7	54 ± 15	43 ± 17
**Hb (mg/dL, *normal F: 12–16, M: 14–18*)**	ND	14 ± 1.5	13 ± 2.2	ND
**Bilirubin (mg/dL, *normal < 1.1*)**	ND	1.08 ± 0.83	1.4 ± 1.07	0.4 ± 0.16
**ALP (IU/L, *normal 30–120*)**	ND	149 ± 68	433 ± 209	82 ± 23
**GGTP (IU/L, *normal F < 66, M < 100*)**	ND	238 ± 116	444 ± 275	18 ± 13
**ALT (IU/L, *normal < 40*)**	ND	193 ± 45	158 ± 42	15 ± 8.4
**Cirrhosis (*yes/no*)**	N/A	0/10	2/10	N/A
**Duration of the disease (*months*)**	N/A	32 ± 12	44 ± 11	ND

Values are given as mean ± SD unless stated otherwise. Abbreviations: PSC, primary sclerosing cholangitis; UC, ulcerative colitis; Hb, haemoglobin; ALP, alkaline phosphatase; GGTP, gamma-glutamyl transferase; ALT, alanine aminotransferase; SD, standard deviation; N/A, not applicable; ND, no data.

## Data Availability

Not applicable.
